# Retention of bar clip attachment for mandibular implant overdenture

**DOI:** 10.1186/s12903-022-02262-7

**Published:** 2022-06-09

**Authors:** Hossam I. Nassar, Medhat Sameh Abdelaziz

**Affiliations:** Department of Prosthodontics, Faculty of Oral and Dental Medicine, Future University in Egypt, Fifth Settlement, End of 90 Street, Cairo New Cairo City, Egypt

**Keywords:** Dental materials, Denture precision attachment, Dental implantation, Denture retention, Computer-aided design

## Abstract

**Objectives:**

The aim of the present study was to evaluate the retention and loss of retention after fatigue testing at different time intervals between two types of bar clip materials (digitally designed PEEK bar clip and regular Nylon bar clip).

**Materials and methods:**

An epoxy model was constructed for a completely edentulous mandible. Two implants were placed according to prosthetically driven implant placement by a computer-guided surgical stent. Bar clips were digitally designed, 3D printed, and pressed into Poly Ether Ether Ketone (PEEK). Pick up of PEEK and nylon clips was performed on the dentures fitting surface using self-cured acrylic resin. Each study group was subjected to an insertion and removal fatigue test simulating 3 years of patient usage. Retention values were recorded using the universal testing machine at initial retention and after 1, 2, and 3 years of simulated usage. For proper sample sizing, 24 models and dentures (12 for each group) were used. An independent sample t-test and repeated measures analysis of variance were used to compare the data.

**Results:**

There were statistically significant differences in retention between the PEEK and nylon bar clips at the beginning of the experiment (*p* = 0.000*). But after 3 years of simulated use, there was no significant difference in retention between the test groups (p = 0.055, NS). After 3 years of simulated use, the retention of PEEK clips decreased by − 58.66% recording 17.37 ± 1.07 N, while the retention of nylon clip increased by + 2.99% recording 16.56 ± 0.88 N.

**Conclusion:**

The digitally designed PEEK clip showed comparable retention results to the nylon clip after 3 years of simulated use.

**Clinical relevance:**

Maintenance of bar attachment with PEEK clip offers a clinical solution after the wear of normal plastic clips, which is a cheap solution that is easily fabricated and picked up into the denture. Digital fabricated PEEK bar retentive inserts can be used in cases of bar attachment wear.

## Introduction

Oral rehabilitation of edentulous and partially edentulous patients has been improved by the development of implants and their different prosthetic options [1]. Several clinical trials have proved that placement of implants in mandibular retained and/or supported overdentures results in a better quality of life compared to conventional complete dentures [1–4].

Implant overdenture can either use splinted implants by bar attachments or un splinted implants by stud-type attachments [5, 6]. Many factors affect appropriate attachment selection, such as jaw morphology, inter arch distance, the desired retention, prosthesis type, inclination and number of implants, patient manual dexterity, financial options, and the availability for maintenance recall visits [7].

Bar attachment is used to splint implants with the lowest complications in the prosthetic superstructure and maximum patient satisfaction [8]. It offers stress-breaking action and cross-arch involvement, which allows occlusal forces to be shared between the abutments [9]. The ideal length of a single bar should range from 20 to 22 mm to accommodate two clips [10]. It also requires an inter-arch distance of a minimum of 15 mm [11].

The bar can be fabricated from metal or milled from a non-metal material such as zirconia and PEEK (Poly-ether-ether-ketone), while the bar clip can be fabricated from PEEK or Poly Oxy Methylene (POM) [12–14]. Polyvinylsiloxane (PVS) has also been introduced and employed as an attachment matrix, which is similar to elastic impression materials, offering a chairside quick solution [15].

PEEK was first developed in 1978 as a thermoplastic, polycyclic, semi-crystalline polymer obtained by binding ketone and ether functional groups with aryl rings [16]. It has superior mechanical properties with resistance to hydrolysis, chemical wear, and high temperatures [17]. It is a biologically inert material with no evidence of cytotoxicity or immunogenicity. It also offers corrosion resistance, low plaque affinity, and minimal creep [14].

In a study by Savabi et al. [18] it was reported that the retention forces for the bar attachments have decreased to (44%) after 5 years of follow-up. Regular bar maintenance depends on the activation of bar clips and even changing the clips [5, 11, 18].

The retention is the first factor responsible for patient satisfaction with the prosthesis, and it is defined as that quality inherent in the dental prosthesis acting to resist the forces of dislodgment along the path of placement [19, 20]. (33%) of prosthodontic complications are related to loss of retention [8]. The rate of attachment wear is related to its material of construction, which should be wear-resistant to maintain a stable retention force overtime. [12]

Burns et al. [39] concluded that the least accepted retention force gained by different attachment systems in implant‑retained overdenture was between 5 and 8 N in the long‑term function [21, 22]. Therefore, studies on the retention of attachments are very important to determine the selection of specific retention systems for the patient [23].

The bar overdenture with PEEK clip has low plaque accumulation with no gingival inflammation with high patient satisfaction in cleaning and maintenance compared to fixed full arch restorations [13]. The PEEK clip represents an alternative to metal ones with favourable prosthetic and clinical outcomes. It has high wear resistance which in turn decreases the number of maintenance visits and the need for changing the clip [14].

### Aim of the study

The objective of the present investigation was to compare digitally designed PEEK bar clips with the well-known nylon clip in their initial retention force and after a simulated 3 years of bar attachment usage. The null hypothesis is that the bar attachment clip fabricated from different materials offers similar retention values in retaining mandibular overdenture.

## Materials and methods

A control model standard was fabricated from a cast obtained from a completely edentulous mandible. Any existing undercuts were blocked. The duplication of the stone model was carried out using laboratory addition silicone material (REPLISIL 22N, dent-e-con, Germany) to obtain an epoxy resin model (Swiss Chem; construction chemicals, Egypt).

For surgical guide construction**,** Cone Beam Computed Tomography (CBCT) (parameters 85 KVP, 5 MA) was recorded for the model to create a DICOM file. The standard tessellation language file (STL file) of the model was also obtained using an intraoral scanner (MEDIT i700; MEDIT Corp). The STL file was superimposed on the DICOM file using the best-fit algorithm.

Using computer-aided design software (Exocad GMBH Dental CAD), a virtual lower denture was designed.

A prosthetically driven implant placement concept was clear in mind, therefore a surgical guide using the virtual designed lower denture was constructed by an implant planning software (real guide; 3diemme, Italy). The implants were placed bilaterally between the lateral incisor and canine. The surgical guide was printed using clear surgical guide resin (EPAX Clear Resin; EPAX 3D). The resultant surgical guide was finished and cleaned with alcohol to remove excess monomer.

**Fig. 1:**
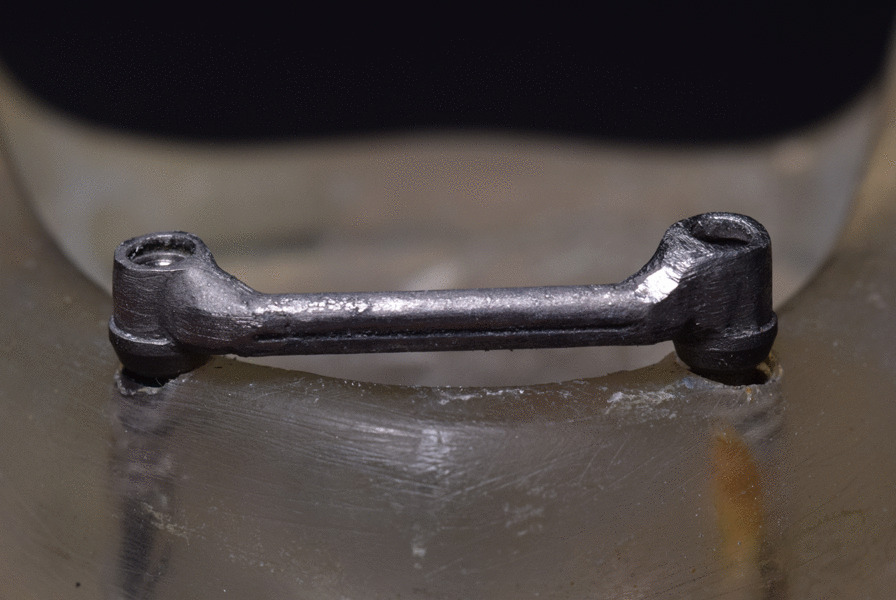
Bar fabricated over the model

Two dental implants (Internal Tapered; BIOHORIZONS) of 3.8 mm in diameter and 10.5 mm in length were loaded into the model. Two castable plastic abutments were tightened using a torque wrench at 25 N over the implants. With the aid of a dental surveyor, the plastic bar with a round cross-section was attached to the two plastic abutments using self-cure acrylic resin, creating a 2 mm clearance space between the bar and the model. The assembly was then unscrewed, cast in Ni–Cr alloy, and finished and polished. The finished bar was checked for passivity and then finally screwed to the implants (Fig. 1).

The model with the bar screwed on was scanned using a desktop scanner (Medit T500) after the application of scan spray powder (Alldent, Germany) to get the STL file of the model and bar. The STL file of the bar and model was imported to the Meshmixer software (MESHMIXER 3.5 software, Autodesk).

**Fig. 2:**
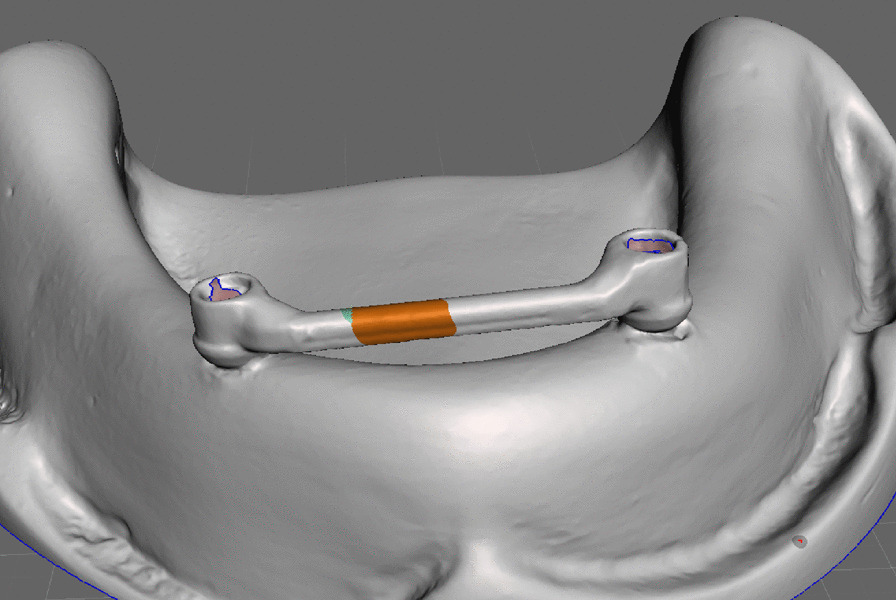
Outlining the PEEK clip

**Fig. 3:**
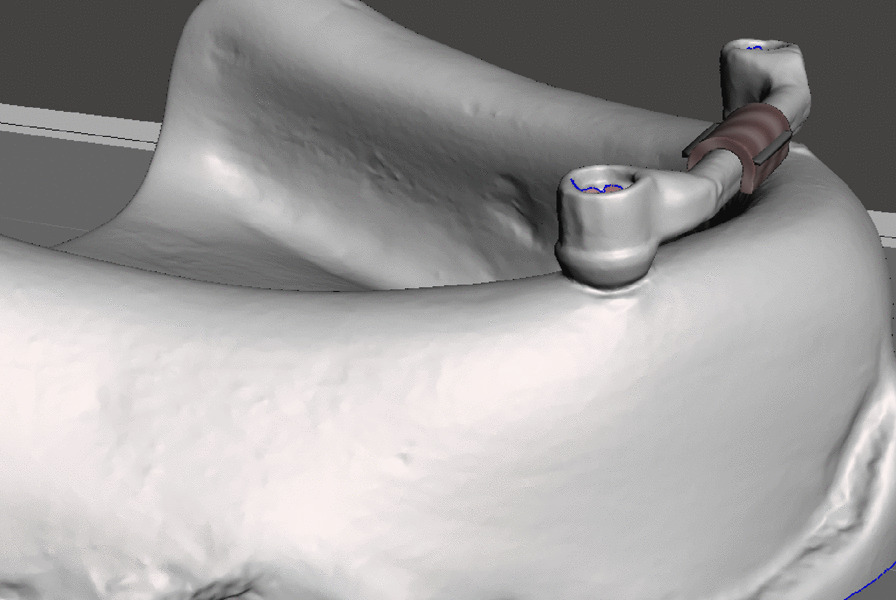
The final for PEEK clip design with an outer surface undercut to provide mechanical retention with the denture

First, the PEEK clip design was drawn on the model by outlining the lingual, buccal, mesial, and distal extents (Fig. 2). The boundaries were smoothened. Undercuts were created on the buccal and lingual aspects of the bar clips by using an attract brush tool to ensure mechanical retention between the clip and the denture fitting surface in the pickup step [24] (Fig. 3).

**Fig. 4:**
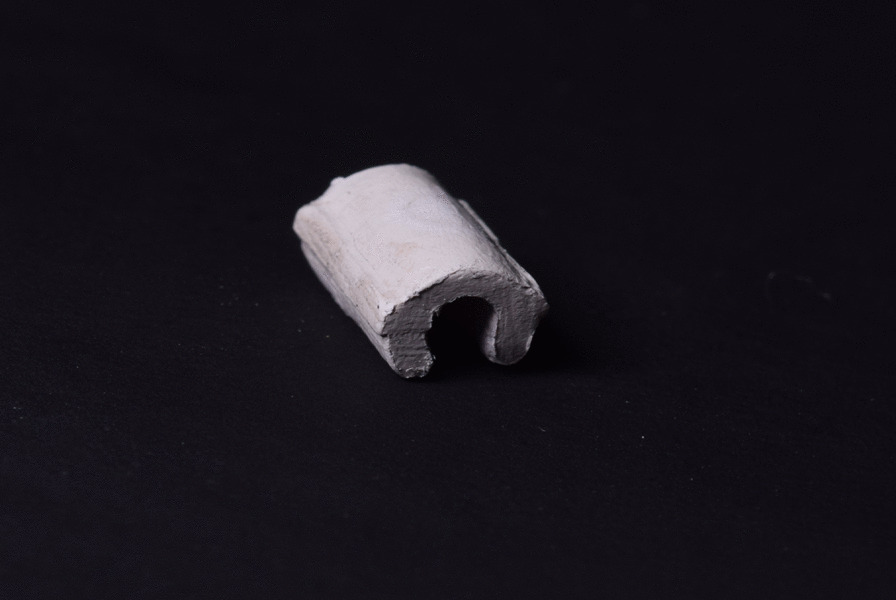
Pressed PEEK clip

The designed clip was printed using (EPAX Dental Castable Resin; EPAX 3D). The clip wax pattern was pressed by the lost wax technique into PEEK (Fig. 4).

The epoxy model was duplicated into the stone cast on which 24 mandibular trial denture bases with waxed-up acrylic resin teeth (Zhengzhou Linker Medical Equipment Co., Ltd.) were fabricated. Mandibular trial dentures were flasked and packed with heat-cured resin (Denture Base Material; Vertex-Dental B.V.), then finished and polished with a hock attached to the denture geometric center.

**Fig. 5:**
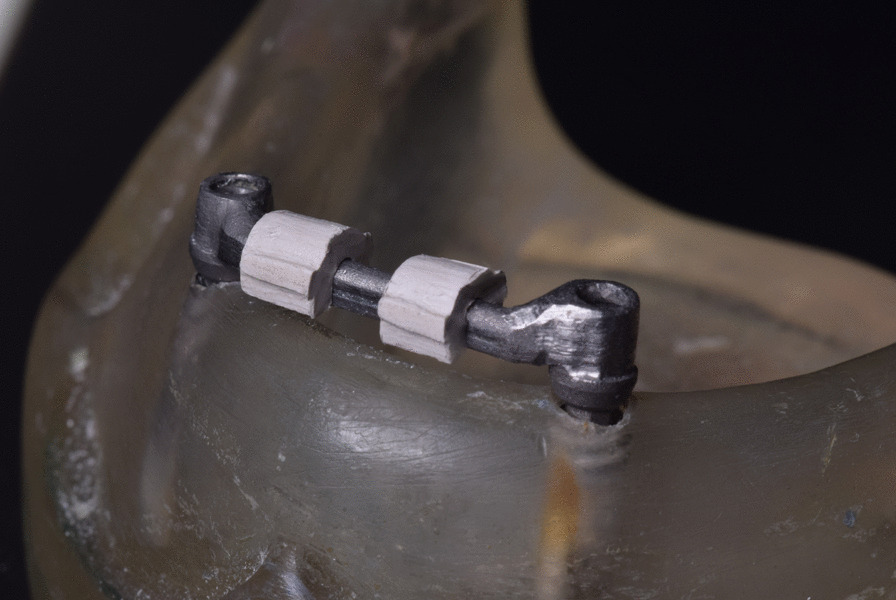
Two nylon clips over the bar for pick up

**Fig. 6:**
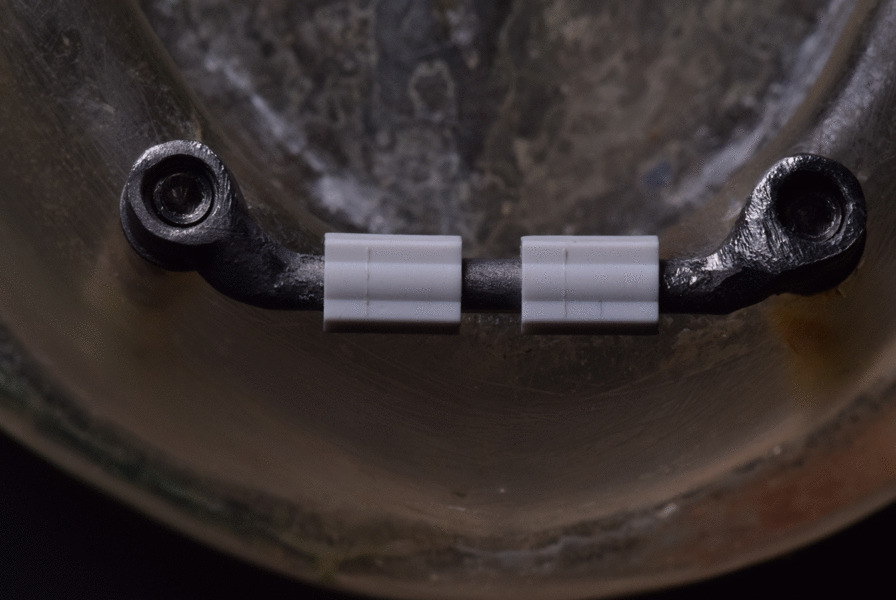
Two PEEK clips over the bar for pick up

The tested groups were classified as follows:

- **Group A** Bar with a plastic (POM) clip attachment (control group) (Fig. 5).
- **Group B** A bar with a PEEK clip attachment (Fig. 6).

A light body rubber base was loaded into the denture, and the denture was tried on the model with a bar and clips loaded on it. Any pressure areas preventing the denture from complete seating or any areas responsible for denture frictional retention were removed. The retromolar pads were used as a reference for the complete seating of the denture base on the model [18].

**Fig. 7:**
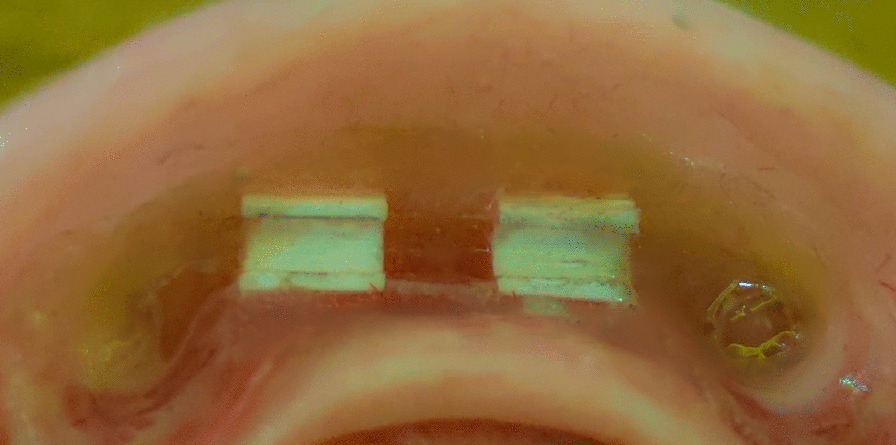
Pick up of the nylon clip in the denture

**Fig. 8:**
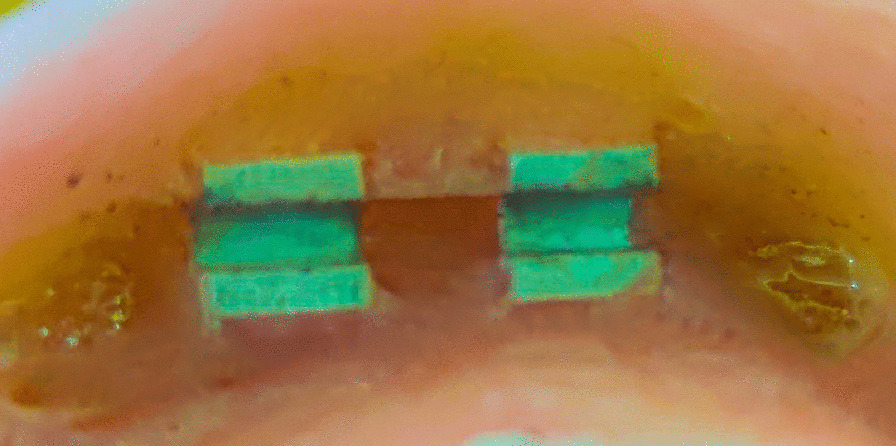
Pick up of the PEEK clip in the denture

Teflon and wax material were used to block any undercuts beneath the bar. Escape holes were made on the denture to act as an exit for the extra pickup material. The pickup was done by self-cured acrylic resin with the denture seated completely over the model. (Figs. 7 and 8).

The specimen’s retention forces were measured using the Instron universal testing machine (model 3345; England). The denture was attached to the dynamic part of the universal testing machine via a screw hook. The direction of the pull forces was performed vertically.

The achieved maximum values of retention force were recorded at the beginning of the study (initial retention) and after 1, 2, and 3 years with an average of 1000 cycles per year based upon patients’ average of 3 insertions and removals per day [22, 25]. Twenty-four epoxy models and dentures were used (12 for each group) for proper statistical sample sizing.

The sample size was calculated using G Power version 3.1.9.2. and according to previous studies [8, 25, 26].

### Statistical methodology

The data was collected and entered into the computer using the SPSS (Statistical Package for Social Science) program (version 21). The data was normally distributed by the Kolmogorov–Smirnov test of normality, so the parametric statistics were adopted. The mean, standard deviation, and 95% CI of the mean were used to describe the data.

Two studied independent, normally distributed variables were compared using an independent sample T-test. Repeated measures analysis of variance was used. Model assumptions were tested and found to be satisfactory except for Mauchly’s test of sphericity.

## Results

**Fig. 9:**
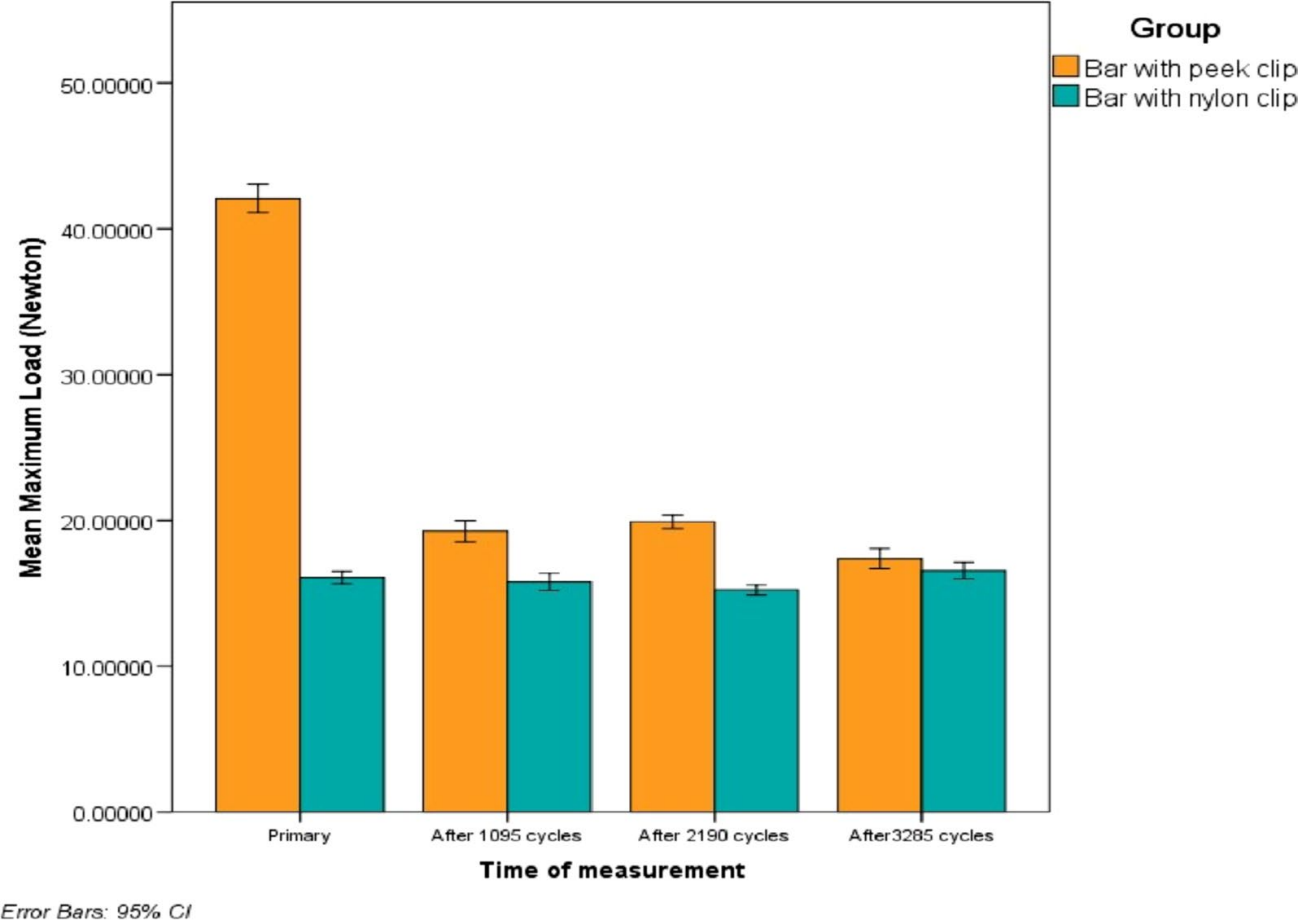
Bar chart of mean retention loss in (Newton) between the two studied groups at a different time of measurement primary retention versus one, two, and three years of use

Comparisons in retention between two studied groups show a statistically significant difference in mean retention at the initial retention test and after different time intervals. (*p* = 0.000*). But after 3 years of stimulated use, there was no significant difference in retention values (p = 0.055 NS) (Fig. 9) (Table 1).

**Table 1:** Retention in (Newton) between the two studied groups at various measurement times

	PEEK clip (M ± SD)	Nylon clip (M ± SD)	*P* value
T0	42.08 ± 1.52	16.08 ± 0.68	0.000*
T1	19.26 ± 1.15	15.78 ± 0.93	0.000*
T2	19.90 ± 0.73	15.22 ± 0.53	0.000*
T3	17.37 ± 1.07	16.56 ± 0.88	0.055 NS

T0, at time of overdenture insertion; T1, after 1 year of use; T2, after 2 years of use; T3, after 3 years of use; NS, Statistically not significant (p ≥ 0.05)

The repeated measure analysis test revealed a statistically significant loss of the mean of retention of the PEEK clip attachment group at different years of use *(p* = 0.000*), with the exception of no statistically significant retention loss between the 2nd and 1st year of testing (*p* = 1.000). For the nylon clip attachment group, the mean of retention between 2 years and primary retention, 3rd, 1st year and 3rd and 2nd year was statistically significant at (p = 0.002), (*p* = 0.006*), and (*p* = 0.000*) respectively (Table 2).

**Table 2:** Repeated measure analysis of variance, comparing retention loss in (Newton) between different time periods of measurement (Primary retention vs one, two, and three years of use) in each studied group

	PEEK clip p value	Nylon clip *P* value
T0–T1	0.000*	1.000 NS
T0–T2	0.000*	0.002*
T0–T3	0.000*	0.195 NS
T1–T2	1.000 NS	0.420 NS
T1–T3	0.000*	0.006*
T2–T3	0.001*	0.000*

T0, at time of overdenture insertion; T1, after 1 year of use; T2, after 2 years of use; T3, after 3 years of use; NS, Statistically not significant (p ≥ 0.05)

*Statistically significant (p < 0.05)

An increase in the percentage of retention for the Nylon clip attachment group after 3 years of use was noticed by + 2.99% compared to primary retention and by + 8.76% compared to 2 years of use. While the PEEK clip attachment group showed an increase in retention after 2 years of use by + 3.72% compared to 1 year of use (Table 3).

**Table 3:** Percentage of retention loss in (Newton) between the two studied groups at various times of measurement Primary retention vs one, two, and three years of use

	PEEK clip (M ± SD) (%)	Nylon clip (M ± SD) (%)	p value
	Percentage change		
T1 versus T0 (%)	− 54.13	− 1.80	0.000*
T2 versus T0 (%)	− 52.63	− 5.24	0.000*
T2 versus T1 (%)	+ 3.72	− 3.28	0.018*
T3 versus T0 (%)	− 58.66	+ 2.99	0.000*
T3 versus T2 (%)	− 12.53	+ 8.76	0.000*

T0, at time of overdenture insertion; T1, after 1 year of use; T2, after 2 years of use; T3, after 3 years of use; NS, Statistically not significant (p ≥ 0.05)

*Statistically significant (p < 0.05)

## Discussion

In completely edentulous patients that need full arch rehabilitation, the presence of some anatomical and surgical limitations can affect the implant placement positions and the number of implants, which necessitates the search for prosthetic solutions with perfect function and aesthetics and load distribution of implants [27]. The bar retained over denture offers a standard of care prosthetic solution by placing two implants in the canine area.

Complete denture digitalization used for implant surgical guide fabrication standardizes clinical results and research work and guarantees implant position according to prosthetically driven implant placement, leading to better load distribution between implants [28, 29].

A round cross-section bar (OT bar multisystem, Rhein83) design was selected in the present study to permit movement of the retained overdenture and allow better occlusal load distribution between implants and residual ridge [13].

The fabrication of the PEEK clip requires optical scanning of the bar attachment, which is more comfortable for patients with less nausea and anxiety compared to conventional impression [30, 31]. With the development of digital dentistry and computer-aided design and computer-aided manufacturing (CAD-CAM) technology, the design of dental attachments and retentive inserts has become easier with perfect expectable results [24, 30].

The designed PEEK clip had an undercut in its polished surface to guarantee mechanical interlocking during pick- up on the intaglio surface of the denture [24].

The wear of retentive clips over bar attachments has been documented to directly influence the retention of overdentures, and attachment wear occurs as a result of friction between retentive attachment surfaces at insertion and removal or during masticatory cycles [20]. Williams et al. [32] reported that the plastic retentive clips, not the round bars, were responsible for the retention loss. For this reason, there is a need to evaluate the retention force of different bar clips if different materials.

The maximum dislodging force was identified as the highest force utilized before the complete separation of attachment components; it could be used as an alternative measurement of overdenture retention and differs with the number of insertions/removal cycles. These tests could enable the clinicians to choose the most efficient attachment system and proper material for each patient. The conventional Instron (IS) testing machine has been well recognized as a reliable and acceptable instrument to test retention forces in vitro [8, 33, 34].

Previous in vitro studies tested the changes in retention force between plastic clips and metal matrices. Plastic clips of poly-oxy-methylene (POM) reported fewer changes in retention compared to metallic ones. This may be attributed to their modulus of elasticity with superior resiliency. Consequently, the plastic clips turned out to be more prevalent and commonly used [33, 35].

Carbon fibers reinforcement is considered one of the recent innovations in the prosthodontics field. It has many applications, such as crowns, bridges, and full arch hybrid restorations. The reinforcement of PEEK with carbon fibers has a great impact on load absorption, resiliency, wear resistance, and patient comfort [36]. Recently, PEEK was introduced as an attachment tool due to its high mechanical properties such as high retention and wear resistance. Abdelrehim et al. concluded that BioHPP (PEEK) bar seems to be a solid candidate for bar fabrication with minimal loss of retention and better wear resistance of the clip [37].

In a clinical 1 year trial, Abdraboh et al. [14] reported that PEEK housing could be an effective alternative to metal housing for a milled bar over inclined implants in the mandible with favourable prosthetic and clinical outcomes. The PEEK housing showed higher satisfaction with retention, stability, speech, and esthetics and a lower incidence of female clip wear and renewal rate [14].

The results of the present study showed a significant difference in the values of initial retention forces where the PEEK clip documented higher retention forces in comparison to the nylon clip. However, an insignificant difference was recorded by the end of the 3 years study period. This result is in accordance with several clinical and in-vitro studies. In their study, Emera and Altonbary reported a significant difference in initial retention forces for the PEEK clip group over the POM clip group, although both clip materials showed no significant difference in retention loss after 1 year of mandibular overdenture clinical use over zirconia bar [13]. In a split-mouth clinical study, Bayer et al. [12] registered significant retention loss with PEEK clips in the first 3 months with a round cross-section metal bar. However, after 6 months, a stabilized retention loss with no statistically significant difference in the clinical performance of PEEK or POM clips was recorded [12].

Hammas et al. [38] concluded that PEEK clips showed more wear resistance than POM clips with metal or PEEK bars, and both materials showed in vitro comparable results concerning retention force [38].

The least reported acceptable retention force of different attachment systems for implant‑retained overdenture ranged between 5 and 8 N throughout the long‑term function. [39] Both studied groups reported higher values by the end of the study period of 3 years. Although results of in-vitro studies that evaluated retention forces of different attachment systems cannot completely mimic all clinical conditions such as exact saliva composition, oral environment, temperature, and patient parafunctional habits that may influence the results, they can point out the performance of the new attachment materials and their gradual retention loss to be used as a substitute for traditional ones.

## Conclusions

With the limitations of the present study, it could be concluded that:

1. Virtually designed and fabricated PEEK clips demonstrated no statistically significant difference in final retention values from nylon clips after stimulated 3 years of use.
2. Application of digital designing of custom-made attachment clips provides ease for overdenture maintenance.
3. PEEK clips could be used as an alternative to nylon and metal bar clips with comparable retention values with resistance to wear and surface alteration.
4. This is an in-vitro study which cannot simulate oral cavity temperature, patient para functional habits, and the effect of different foods and beverages with either acidic or alkaline composition on the wear of different clip materials.
5. It is recommended to perform clinical ling term in vivo study for custom made PEEK clip attachments. It is also recommended to test the attachment performance with different denture cleansing solutions and to test the PEEK clip with different percentages of glass fiber reinforcement to improve its wear resistance.

## Data Availability

The datasets used and/or analysed during the current study are available from the corresponding author on reasonable request.
